# Radiological outcomes of PEEK rods in patients with lumbar degenerative diseases: A minimum 5-year follow-up

**DOI:** 10.3389/fsurg.2023.1146893

**Published:** 2023-03-27

**Authors:** Weimin Huang, Wenqiao Wang, Xiaoduo Xu, Lei Wang, Jingming Wang, Xiuchun Yu

**Affiliations:** Orthopaedic Department, 960 Hospital of People’s Liberation Army, Jinan, China

**Keywords:** PEEK rods, lumbar degenerative diseases, radiological outcomes, rehydration, hybrid, non-fusion

## Abstract

**Purpose:**

To determine the long-term radiological outcomes of PEEK rods in patients with lumbar degenerative diseases.

**Methods:**

Radiological outcomes of cohort cases with lumbar degenerative diseases following PEEK rods were retrospectively studied. Disc height index (DHI) and range of motion (ROM) were measured by x-rays. The CT scans and reconstruction were used to determine screw breakage, rods fracture, screw loosening and intervertebral bony fusion status. The MRI scans were used to evaluate the changes of intervertebral discs at the non-fusion segments and adjacent segments in terms of Pfirrmann Classification.

**Results:**

A total of 40 patients completed the mean of 74.8 ± 9.6 months follow-up, with 32 patients undergoing hybrid surgery and 8 patients undergoing non-fusion surgery. The mean DHI changed from preoperative 0.34 to 0.36 at the final follow-up and the ROM declined from 8.8° preoperatively to 3.2° at the final visit, however, both had no statistical differences. Of the 40 levels underwent non-fusion procedure, 9 levels showed disc rehydration with 7 patients from Grade 4 to Grade 3 and 2 patients from Grade 3 to Grade 2. The other 30 cases did not show distinctive change. No screw loosening or rods breakage were detected during the follow-up periods.

**Conclusion:**

PEEK rods have obvious protective effects on degenerated intervertebral disc of non-fusion segments and the incidence of complications related to internal fixation is low. PEEK rods pedicle screw system is safe and effective in the treatment of lumbar degenerative diseases.

## Introduction

Low back pain has been reported to be the leading cause of disability worldwide (1). In general, the symptoms can be significantly relieved by conservative treatment. However, a fusion procedure may be considered in those patients with persistent symptomatic degenerative disc disease, such as lumbar disc herniation, lumbar spondylolisthesis and lumbar spinal stenosis (2). In the past decades, lumbar interbody fusion aided by titanium rods and pedicle screws has been widely used (3). With its increasing application, related complications like adjacent segment degeneration, pseudoarthrosis and hardware failure have also raised great concerns (4, 5).

Attempts have been made to modify the rods for the purpose of reducing these complications. Various novel semi-rigid or dynamic internal fixations such as Dynesys, ISObar-TTL, Bioflex have been designed and applied clinically (6–8). Different from these devices, PEEK (Polyetheretherketone) rods are an innovation of materials. PEEK materials have offered broad applications in the joint prosthesis, intervertebral cages, meshes, et al. (9). Due to the lower elastic modulus compared with titanium rods, posterior stabilization with PEEK rods may offer optimized stress attribution (10). Previous studies have demonstrated that PEEK rods more closely approximated the physiologic anteroposterior column load sharing compared with titanium rods (11).

Despite the biomechanical advantage, preliminary clinical outcomes seem to be inconsistent. Some studies reported satisfactory clinical effects, while others reported high reoperation rates (12–14). Besides, rare information is available on the long-term outcomes, especially regarding radiological outcomes. In addition, some previous studies have exhibited disc rehydration induced by posterior lumbar dynamic fixations, however, no similar observation have been reported on PEEK rods (15, 16). Therefore, the current study was undertaken to uncover the radiographic outcomes of PEEK rods *in vivo* with a minimum 5-year follow-up.

## Methods

### Study design

From October 2013 to March 2016, a consecutive series of patients who underwent surgical treatment of lumbar degenerative diseases with PEEK rods were retrospectively collected following local Ethics Committee approval. Informed written consent were obtained for all the enrolled patients. Demographic characteristics, radiographic parameters, clinical outcomes and complications were reviewed. Hybrid procedure in this study means both fusion procedure and non-fusion procedure in one patient.

 Inclusion criteria: (1) Patients with a clear diagnosis of symptomatic lumbar degenerative diseases such as lumbar spinal stenosis, lumbar degenerative spondylolisthesis, lumbar disc herniation; (2) Patients who have failed conservative treatment for more than three months; (3) Patients who underwent PEEK rods internal fixation with non-fusion or hybrid procedure; (4) Patients who were followed up for more than 5 years.

Exclusion criteria: (1) Those with a previous history of lumbar spine surgery; (2) Those with isthmic spondylolisthesis or lumbar spondylolisthesis of degree II or above; (3) Those with lumbar trauma, infection, tumor or severe osteoporosis; (4) Those with incomplete follow-up data.

### Surgical procedure

After general anesthesia, patients were placed prone with appropriate positioning precautions. A standard midline incision with typical exposure procedure was made. Attention should be paid to preserve the integrity of the supraspinous and interspinous ligaments. After confirming the index segments by C-arm, pedicle screws were inserted. Laminectomy, partial laminotomy, complete or partial facet resection were made to ensure sufficient decompression of the dural sac and nerve roots. Facet joints were preserved as far as possible as previous research suggested (14). For those index levels requiring discectomy, intervertebral fusion procedures with PEEK cages were applied. Then proper size PEEK rods (Wego, Shandong, China) were placed bilaterally and a spinal distraction was provided at the non-fusion level for distracting the disc space. After ascertaining all the screws in good position by fluoroscopy, a drainage tube was placed and the incision was closed layer by layer. Patients were ambulatory right after the removal of drainage tubes.

### Clinical evaluation

Clinical outcomes were evaluated by visual analog scales (VAS) and the Oswestry Disability Index (ODI) preoperatively and at the final follow-up.

### Radiological analysis

The standard anterior-posterior, lateral and flexion-extension fluoroscopy of the lumbar spine, the CT scans with 3D reconstruction and the MRI scans preoperatively and at the final follow-up were obtained and analyzed.

**Figure 1:**
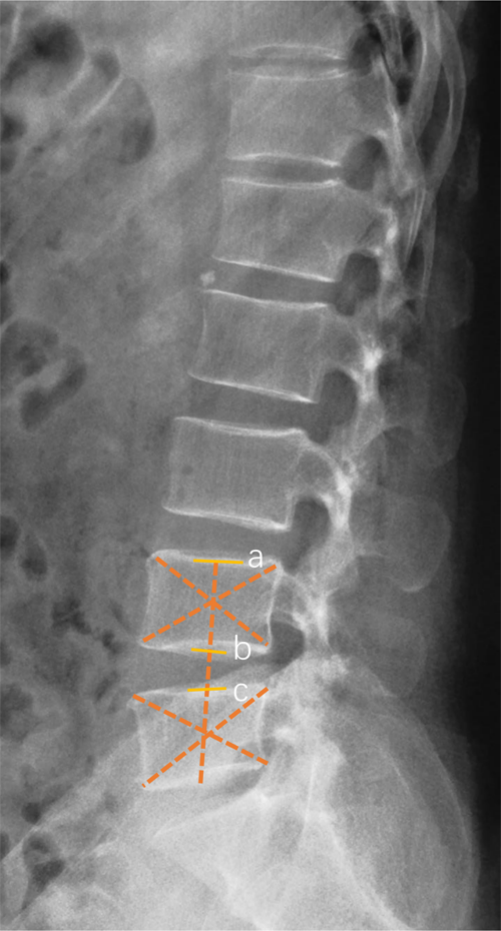
Schematic diagram of intervertebral disc height index (DHI) measurement. DHI was the ratio of intervertebral disc height (bc) to upper vertebral body height (ab).

The disc height index (DHI) (Figure 1) and range of motion (ROM) were measured on the x-ray film. Lumbar lordosis was evaluated in terms of the lumbar lordosis angle (LLA). Screw breakage could be detected by axial scanning and sagittal reconstruction. Screws loosening was defined as a radiolucent zone around the implant (17). Due to its radiotransparency, the integrity of PEEK rods was observed by two or three-dimensional CT reconstructions. Bony fusion was defined as continuous trabecular bone through the adjacent vertebral endplates on the two-dimensional CT reconstruction (18). Disc degeneration of the non-fusion segment and adjacent segment was an evaluation by Pfirrmann Classification on the MRI scans (19).

### Statistical analysis

Statistical analyses were conducted using SPSS version 17.0. Continuous variable comparisons were conducted using the paired sample t-test. *P*-values of less than 0.05 were considered to have statistical significance. The Cohen's kappa values were calculated for intra-observer reliability and the Fleiss kappa values were calculated for interobserver reliability between the two observers. The Landis and Koch interpretation of kappa values was used.

## Results

### Baseline characteristics

**Figure 2:**
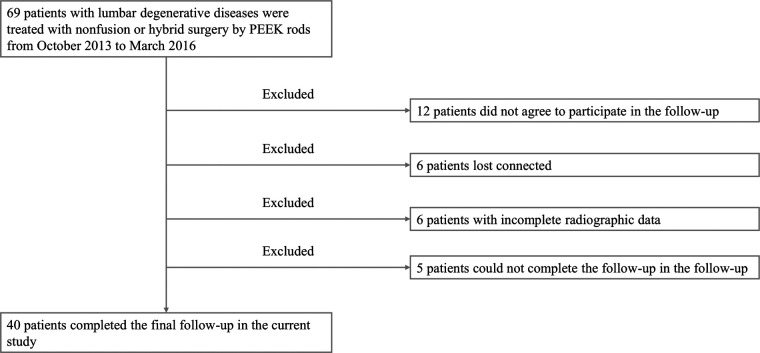
Flow chart of patient selection process.

A total of 69 patients with posterior hybrid or non-fusion surgery by PEEK rods were collected in the current study. After excluding those who did not agree to participate in the follow-up, those who lost contact, and those with incomplete radiographic data, there were 40 patients returning for the final follow-up measurements. The detailed patient selection process was showed in Figure 2. Of these 40 patients, 23 were females and 17 were males. The mean age was 54.5 years old ranging from 29 to 74 years old. The mean BMI was 25.7 kg/m^2^ ranged from 17.2 kg/m^2^ to 33.6 kg/m^2^. The follow-up duration ranged from 60 months to 89 months, with a mean value of 74.8 months. Baseline characteristics were demonstrated in Table 1.

**Table 1:** Baseline characteristics of patient.

Characteristics	Patient population
Mean age (SD), years	54.5 (11.2)
Range	29–74
**Gender, *n* (%)**
Male	17 (42.5%)
Female	23 (57.5%)
BMI(SD), kg/m^2^	25.7 (3.6)
Range	17.2–33.6
**Smoker, *n*(%)**
Nonsmoker	32 (80.0%)
Smoker	8 (20.0%)
**Diabetes**
Yes	6 (15.0%)
No	34 (85.0%)
Follow-up duration (SD), months	74.8 (9.6)
Range	60–89
**Diagnosis, *n* (%)**
Lumbar spinal stenosis	15 (37.5%)
Lumbar spondylolisthesis	10 (25.0%)
Lumbar disc herniation	15 (37.5%)
**Surgical procedure, *n* (%)**
**Hybrid surgery**
L4/5 fusion, L3/4 nonfusion	18 (45.0%)
L5/S1 fusion, L4/5 nonfusion	6 (15.0%)
L3/4 fusion, L4/5 nonfusion	3 (7.5%)
L4/5 fusion, L5/S1 nonfusion	4 (10.0%)
Other procedure	1 (2.5%)
**Nonfusion procedure**
One level	7 (17.5%)
Two levels	1(2.5%)

SD, standard deviation.

### Radiographic assessment

All the enrolled patients completed the standard anterior-posterior and lateral fluoroscopy of the lumbar spine and 37 patients had flexion and extension fluoroscopy. The intraobserver reliability showed moderate and substantial agreement (ƙ = 0.59 and ƙ = 0.68), and the interobserver reliability showed substantial agreement (ƙ = 0.65).The mean lumbar lordosis angle at the non-fusion levels changed from a preoperative mean of 13.2° to 11.4°at the final follow-up with statistical significance. Regarding lumbar lordosis, no statistical difference was observed when comparing preoperative values with the final follow-up. The DHI at the non-fusion level changed from 0.34 preoperatively to 0.36 at the final follow-up without statistical significance. The ROM of the non-fusion level declined from 8.8° preoperatively to 3.2° at the final visit still without a statistical difference (Table 2).

**Table 2:** Radiographic outcomes.

Radiographic assessment	Preoperative mean value, (SD, range)	Postoperative value at the final follow-up, (SD, range)	*P* value
Lumbar lordosis angle at the index level	13.2° (8.9°, 0.6°–42.0°)	11.4° (8.2°, 0.5°–35.0°)	*P* = 0.000 < 0.05
Lumbar lordosis	37.5° (10.5°, 9.0°–58.7°)	38.8° (11.1°, 13.1°–64.0°)	*P* = 0.376 > 0.05
Disc height index	0.34 (0.13, 0.11–0.61°)	0.36 (0.11, 0.16–0.66)	*P* = 0.283 > 0.05
Range of motion	8.8° (3.1, 4.3°–17.0°)	3.2° (1.6, 0.7°–6.0°)	*P* = 0.412 > 0.05

SD, standard deviation.

Of the 32 patients who underwent hybrid surgery, bony fusion was confirmed in 27 patients. The bony fusion rate was 84.4%.

**Figure 3:**
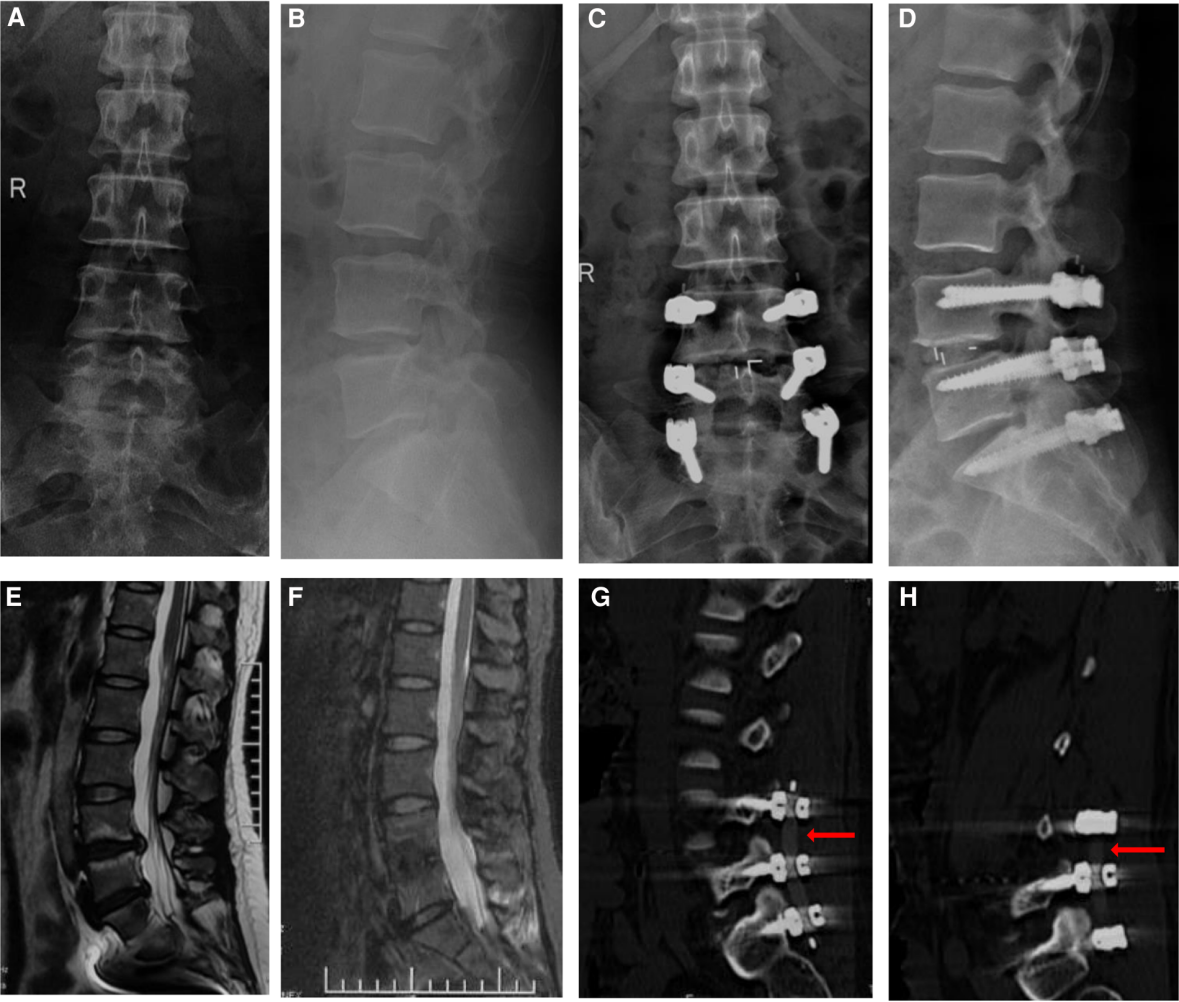
A 45 years female patient diagnosed with lumbar spinal stenosis underwent posterior PEEK rods hybrid surgery with fusion procedure at the L4/5 level and non-fusion procedure at the L5/S1 level. Preoperative anterior-posterior and lateral fluoroscopy was showed in (**A**,**B**), while postoperative anterior-posterior and lateral fluoroscopy at the final follow-up was showed in (**C**,**D**). Compared preoperative sagittal MRI scans (**E**) with sagittal MRI scans at the final follow-up (**F**), it was found that intervertebral disc degeneration improved from Grade 4 to Grade 3 according to Pfirrmann Classification. The CT reconstruction confirmed that the bilateral PEEK rods were intact at the final follow-up (**G**,**H**).

A total of 40 levels underwent a non-fusion procedure including the non-fusion levels in the hybrid group and non-fusion levels in the non-fusion group. The MRI scans at the final follow-up demonstrated a significant change that 9 of the 40 levels showed disc rehydration. Of the 9 patients, 7 patients improved from Grade 4 to Grade 3 and 2 patients improved from Grade 3 to Grade 2 according to Pfirrmann Classification (Figure 3). 1 case showed disc degeneration from Grade 3 to Grade 4. The other 30 cases did not show a distinctive change in the intervertebral discs on the MRI scans.

Concerning adjacent segment changes, there were 14 patients out of the 40 patients showed adjacent segment degeneration (ASD) based on the MRI scans. Of the 14 patients, 4 patients changed from Grade 4 to Grade 5, 6 patients from Grade 3 to Grade 4, 2 patients from Grade 2 to Grade 3 and 2 from Grade 2 to Grade 4. Besides disc degeneration, the most common degeneration change at the adjacent segment was spinal canal stenosis caused by epidural fat deposition and ligamentum flavum hypertrophy.

### Clinical outcomes

The ODI score changed from a preoperative mean of 77.5 ± 12.2 to 13.2 ± 10.9 at the final follow-up. The back pain VAS score changed from preoperative 7.9 ± 1.3 to 1.8 ± 1.4 at the final follow-up and the leg pain VAS score changed from 5.9 ± 2.4 to 1.3 ± 1.5. Both ODI score change and VAS score change had statistical differences.

### Adverse events and complications

No related adverse events were recorded during the follow-up periods. There were no wound healing disturbances and no revision surgery. Pedicle screw breakage was observed in one patient without clinical unwell. No screw loosening and migration were detected at the final follow-up. The CT reconstruction confirmed that all the PEEK rods were intact, and no rod breakage occurred during the follow-up.

## Discussion

The current study demonstrated that PEEK rods non-fusion and hybrid surgery had favorable radiographic outcomes during minimal 5 years follow-up. The most significant clinical finding was that 22.5 percent of (9 out of 40) patients exhibited lumbar disc regeneration, which manifested as disc rehydration on the MRI scans.

Disc degeneration may be caused by trauma and chronic stain and is considered as the initial factor of lumbar degenerative diseases (20). Generally, disc degeneration progresses with age. Numerous studies have sought to investigate the mechanism of disc degeneration, however, few effective clinical treatments can be applied to date (21). Disc rehydration has not just been reported in posterior lumbar surgery with the PEEK rods, previous studies have also reported this phenomenon in other posterior lumbar dynamic fixations. Yilmaz has reported 59 patients with lumbar segmental instability treated with Dynesys dynamic fixation. Twenty patients (33.9%) have exhibited disc rehydration (15). Canbay et al. have reported 27 patients with lumbar degenerative diseases treated by the Cosmic dynamic screw system, 4 patients observed disc degeneration improvement (16). Zagra et al. have studied the clinical application of a novel posterior lumbar dynamic fixation called Flex-Plus Spinal System. The MRI images at the 12 months follow-ups demonstrated that 25% (8 out of 32) of degenerative discs improved from Grade 4 to Grade 3 (22). Besides dynamic pedicle screw fixations, interspinous spacers have also been reported to contribute to disc regeneration. Jiang et al. have exhibited a minimum 5 years follow-up of multi-segmental lumbar degenerative disease treated by Wallis interspinous spacer. Of the 26 cases, 4 cases exhibited disc rehydration (23).

It was proposed that distraction or stabilization by these dynamic fixation systems might provide suitable conditions for possible regeneration (24). Our previous studies have investigated the mechanism of tensile stretch in regulating the function of nucleus pulposus cells (25). Nucleus pulposus cells were isolated and cultivated from the lumbar disc tissues obtained from patients who underwent percutaneous endoscopic discectomy. Following the application of cyclic tensile stress of 0.1 Hz for 8,640 cycles, the nucleus pulposus cells demonstrated a significantly greater growth rate, and more nucleus pulposus cells transited from the S phase to the G2/M phase. Moreover, it was noted that the tensile stretch also altered the expression of 31 genes involved in the ITGA2/PI3K/AKT pathway and remarkably promoted this pathway in nucleus pulposus cells.

Previous animal studies have also explored the potential mechanism. Kroeber has investigated the effects of dynamic traction on the disc of rabbit models. After 28 days of compression loading, it was found that the intervertebral disc height significantly decreased, the structure of the nucleus pulposus was disordered, and cell apoptosis significantly increased. The mechanical traction was applied to the rabbit discs, and it was found that the apoptosis of intervertebral disc cells was significantly reduced, and the secretion of proteoglycans in the cytoplasm of cells increased, which indicated the recovery of degenerated rabbit intervertebral discs (26). Kuo et al. conducted a biomechanical experiment on a porcine model. A total of 48 thoracic porcine spine models were divided into intact, degeneration and degeneration with traction groups. It was exhibited that straightened collagen fibers increased within the degraded annulus fibrosus, and the annulus pores were less occluded. It was concluded that disc distraction contributed to increase nutrition supply and upgrade disc cell proliferation of the degeneration discs (27). Guehring et al. have established a rabbit lumbar spine model. MRI scans demonstrated the signal intensity decreased when acting 28 days compressive load and then the signal intensity was reestablished following mechanical stretch stress. It was proposed that mechanical distraction promoted extracellular matrix gene expression and facilitated absorption of nutrients into the disc (28). Until now, the mechanism of rehydration of degenerated intervertebral discs still warrants more research and this may provide potential new approaches to lumbar degenerative disc diseases.

Posterior dynamic or semi-rigid pedicle screw fixations once were promising techniques, however, the high revision surgery rate caused by hardware failure has inhibited their clinical applications. Previous studies have reported rod breakage of dynamic pedicle screw systems (29, 30). Therefore, many clinicians are concerned about the integrity of PEEK rods during long-term follow-up. In the current study, no rod breakage occurred during the follow-up period. Zhao has retrospectively examined a cohort of 28 patients who underwent hybrid surgery with PEEK rods. Within the two years follow-up, no screw loosening, rod breakage or other mechanical complications were observed. Some suggestions have been offered to prevent potential rod breakage. First, it has been presented that the rod breakage was more likely to occur in the position of the nut indentation. For PEEK rods, the depth of the nut indentation is mainly dependent on the torque applied to the rods. The PEEK rods system is designed to have a preset torque using a self-breaking nut cap. It should be noted that this design was based on the premise of using counter wrenches (11). If the counter wrench is not used, it may lead the nut cap to need more torque to break and lock, which may lead to deeper indentation of the rod and more likely to break. Second, attention should be paid to make the screws in a good arrangement. Previous studies have reported that PEEK rods have a higher risk of breakage when subjected to shear force (11). As a result, the position of the screw tails should be inserted in a line on the coronal plane and in an arc on the sagittal plane to minimize the shear force possibly.

Another concern about PEEK rods is implant fatigue. Mechanical tests *in vitro* showed that PEEK rods have good fatigue strength (11), but rare information is available when *in vivo*. Although fatigue tests could not be taken after implantation, radiographic outcomes helped make an assessment. It was indicated that PEEK rods had a good fatigue strength *in vivo* and that the DHI was well preserved. The fatigue PEEK rods could not offer sufficient distraction force and disc height support. So it could be determined that the PEEK rods carried favorable duration during the long-term follow-up.

Several limitations should be noted in the current study. First, although as described in the previous section that traction tension is helpful for rehydration of degenerated intervertebral discs (25), it has been reported that excessive traction tension may cause intervertebral disc degeneration. The current study did not provide a standard for traction force, it mainly depended on the clinical experience of surgeons. Second, previous studies have shown that the incidence of ASD was related to the stiffness of internal fixation (31). PEEK rods are considered to be able to prevent ASD potentially due to their lower elastic modulus than titanium rods. The incidence of ASD in this article is 35.0%. Due to the lack of a control group, whether PEEK rods could reduce the incidence of ASD was inclusive in the current studies. Besides, the age range of the selected patient are relatively wide, which may cause a selected bias. This might be settled by a larger sample of control studies in the future.

## Conclusion

PEEK rods have obvious protective effects on degenerated intervertebral disc of non-fusion segments and the incidence of complications related to internal fixation is low. PEEK rods pedicle screw system is safe and effective in the treatment of lumbar degenerative diseases.

## Data Availability

The raw data supporting the conclusions of this article will be made available by the authors, without undue reservation.
